# Editorial: The Cognitive Underpinnings of Anthropomorphism

**DOI:** 10.3389/fpsyg.2019.01539

**Published:** 2019-07-02

**Authors:** Gabriella Airenti, Marco Cruciani, Alessio Plebe

**Affiliations:** ^1^Department of Psychology, University of Turin, Turin, Italy; ^2^Department of Information Engineering and Computer Science, University of Trento, Trento, Italy; ^3^Department of Cognitive Science, University of Messina, Messina, Italy

**Keywords:** anthropomorhism, imagination, theory of mind (ToM), social cognition, human-animal relationship, social robotics, cognitive science of religion

Human beings frequently attribute anthropomorphic features, motivations and behaviors to animals, artifacts, and natural phenomena. Historically, many interpretations of this attitude have been provided within different disciplines (Guthrie, 1993). The attitude of treating artifacts or animals as if they were humans occurs very early in life appearing to be a fundamental aspect of human cognition (Epley et al., 2007; Dacey, 2017). In this Research Topic we set out to investigate some aspects of this phenomenon that are debated in contemporary research in cognitive science.

A first issue concerns how anthropomorphism is acquired and what is the relationship between adults and children's manifestations of this phenomenon. Can we still subscribe to Piaget's view that described animism as a typical children's form of thought (Piaget, 1926/1929)? Is there a relationship between anthropomorphism and pretense and role play? Connected to this there is the question whether anthropomorphism is the product of beliefs—and then linked to human-likeness or assumed complexity of an object or an animal—or instead can be observed only in the context of specific interactions.

Airenti in her paper discusses the acquisition of anthropomorphism in pretend play. She challenges two common views, that everyday forms of anthropomorphism are grounded in beliefs systems and that children would be more prone to anthropomorphism than adults. She argues that anthropomorphism is instead a form of communicative interaction in which a non-human entity takes the place that is generally attributed to a human interlocutor, a format implying the automatic attribution of mental and affective states.

The relation between role play and anthropomorphism in children is the central topic of the work of Severson and Woodard. In their study they analyze individual differences in role play and anthropomorphism in children 5, 7, and 9 years old. Their results provide evidence for a positive relation between the tendency to engage in role play and the tendency to anthropomorphize. They argue that role play and anthropomorphism potentially rely on a common simulation process of imagining others' minds and internal states.

Servais criticizes the definition of anthropomorphism as the attribution of human characteristics to a non-human being, proposing instead a pragmatist view of anthropomorphism. Based on anthropological and ethological literature she analyzes different forms of human-animal interactions, both in everyday life and biomedical laboratories. This evidence shows that anthropomorphism is not the attribution of human qualities to an animal according to a similarity gradient but the situated direct perception of animal minds by someone who is engaged in a specific interaction with them.

Another issue, which is considered in studies on anthropomorphism is how individual variability manifests (Waytz et al., 2010).

Shaman et al. try to determine the underlying structure of individuals' anthropomorphic concept of God, whether there are cultural and experiential predictors of that structure, and whether individuals are consistent in how they anthropomorphize concepts of God in three domains. They assess individuals' attribution of anthropomorphic properties to God in the psychological, biological, and physical domain. They propose an analysis of how these domains relate to one another and an exploration of the experiential and personal factors that contribute to individual differences in anthropomorphizing across these three domains.

A particularly interesting case of difference in the practice of anthropomorphism can be found in clinical groups. Atherton and Cross review the literature about theory of mind and anthropomorphism in relation to individuals with Autism Spectrum Disorder (ASD). From their analysis it appears that ToM abilities which are usually impaired in this population, may be ameliorated, spared, or even enhanced when they are directed toward anthropomorphic rather than human agents. Evidence suggests that individuals with ASD may find anthropomorphic stimuli more socially motivating than human stimuli. This finding leads the authors to conclude that engagement with anthropomorphic stimuli may be used to enhance ToM abilities in this population.

Scientists are no exception, they are as inclined to anthropomorphism as lay people. Therefore, it is worth investigating the effect of anthropomorphism in the scientific practice. Varella identifies three distinct stances underlying mental anthropomorphism in action within biological sciences: the design stance, the basic-goal stance and the belief stance. For example, the design stance may be responsible for the mistaken conviction that function is the only explanation for why traits evolve. By adopting the belief stance the evolutionary gene's point of view is equated to human personal intention. Varella is particularly concerned with misunderstanding about natural selection by biology students caused by anthropomorphism.

Bruni et al. are less worried about the implication of anthropomorphism in the scientific research. Even if anthropomorphism is inherently a logical mistake, they argue that the use of humans as a model in scientific explanation has heuristic advantages, both in everyday circumstances and in the scientific enterprise. Ground for this claim is found in several animal studies, where a careful application of anthropomorphism has led to important discoveries.

Finally, a present theme of debate is the role of anthropomorphism in the design and management of robots and artifacts in general.

Damiano and Dumouchel propose a critical ethical approach to social robotics, which aims at allowing humans to use social robots for self-knowledge and moral growth. They take position in the debate, not only developing a series of arguments relevant to philosophy of mind, cognitive sciences, and robotic AI, but also asking what social robotics can teach us about anthropomorphism. They propose a theoretical perspective that characterizes anthropomorphism as a basic mechanism of interaction, and rebuts the ethical reflection that a priori condemns anthropomorphism-based social robots.

A second contribution in the “applied anthropomorphism” domain is due to Lee et al., demonstrating that anthropomorphism as a design philosophy can have a wide range of applications. The technological object of their study is a flexible display, and they found that the shape of the bend display enables emotional interaction with the users. Unlike the five standard emotions of facial expressions, the device elicited three groups of emotions: happiness, sadness-fear and anger-disgust. Moreover, only a few of the possible shapes of the device evoked high emotional responses.

## Author Contributions

All authors listed have made a substantial, direct and intellectual contribution to the work, and approved it for publication.

### Conflict of Interest Statement

The authors declare that the research was conducted in the absence of any commercial or financial relationships that could be construed as a potential conflict of interest.
